# The complement pathway and the pathophysiology of fibroproliferative cutaneous scarring

**DOI:** 10.3389/fimmu.2025.1701998

**Published:** 2025-10-31

**Authors:** Ilja L. Kruglikov, Katarzyna Walendzik, Philipp E. Scherer

**Affiliations:** ^1^ Scientific Department, Wellcomet GmbH, Karlsruhe, Germany; ^2^ Touchstone Diabetes Center, University of Texas Southwestern Medical Center, Dallas, TX, United States

**Keywords:** complement pathway, fibroproliferation, scarring, *S. aureus*, complement factor H

## Abstract

Fibroproliferative cutaneous pathologies such as hypertrophic scars and keloids have a high prevalence after burns and surgical interventions; however, their pathophysiology remains not fully understood. Here, we formulate a new pathophysiology of cutaneous scarring based on the primary involvement of the dysregulated complement pathway. This pathway is activated in skin wounds to promote their closure and is directed, among others, against *S. aureus*, bacteria that are always present at the site and time of injury. Under some conditions, this can lead to intensive proliferation of *S. aureus*, changing the status of these bacteria in the skin from commensal to pathogenic. Pathogenic *S. aureus* recruits complement factor H (CFH) – a key recognition molecule for the host-*vs*-target identification - to its surface to evade the immune system. This provides an effective suppression of the CFH level in the wound and increases the probability of the production of membrane attack complexes (MACs). The production of MACs can cause an enhanced autolysis of the host cells. This is counter-productive in wound closure. Defensive mechanisms are activated in these cells. One of them is the CAV1-dependent endocytosis that effectively eliminates MACs from cell membranes. This consistently leads to a reduction of the CAV1 content in affected skin areas, causing the established hallmark of hypertrophic scarring (HTS) and keloids (KE), as well as overexpression of RUNX2, which promotes the formation of the cartilage-like hyalinated scar tissue. According to this pathophysiology, future efforts in the prevention and treatment of scarring should be concentrated on the reduction of bacterial content in the wound in combination with a proper modulation of the complement pathway and CAV1 in wounded skin.

## Introduction

Hypertrophic scars (HTS) and keloids (KE) are fibroproliferative cutaneous pathologies resulting from excessive deposition of extracellular matrix at the site of a skin injury, such as burns, surgery or trauma. Prevalence of HTS after full-thickness burns was assessed to be up to 67% (1), which makes this pathology a common condition after such injuries. Different factors were phenomenologically considered to be involved in HTS formation; among others, delayed wound healing with prolonged inflammation, wound infection, and increased mechanical tension at the site of skin damage.

Activation of the complement pathway, including its terminal part, is an important mechanism of the innate immune response to tissue injury that can significantly influence the healing process. Different complement factors are activated during the progression of wound healing. Inhibition of the complement pathway was considered as a target for the improvement of non-healing wounds (2). On the other hand, early topical application of the complement factor C3 or C5 to acute rat skin incision wounds resulted in a dramatic increase of the maximal wound-breaking strengths as well as enhanced production of fibronectin and COL1 compared to control lesions (3). This could reflect the fact that the complement system plays an opposing role in acute *vs.* chronic wounds.

While inflammatory and immunological factors are recognized to play a crucial role in pathological cutaneous scarring, the aberrant activation of the complement pathway was not really considered as the main pathophysiological factor in fibroproliferative cutaneous pathologies. KE tissues, but not normal skin from the same patients, contain complement factors C1q and C3, as well as immunoglobulins (4). Moreover, while KE contain both pro-inflammatory M1 and pro-fibrotic M2 macrophages, the M2 phenotype is highly prevalent. This is similar to the prevalence of macrophages observed in different tumors in which such a polarization of macrophages was linked to the activation of the complement pathway, since the anaphylatoxin receptors C3aR and C5aR are predominantly present on M2 macrophages (5). At least C5aR is strongly involved in the induction of the M2 phenotype (6). It was reported that complement factor H (CFH) plays a crucial role in scarless wound healing and that ectopic application of CFH accelerates wound closure and induces regeneration in typically fibrotic wounds (7).

Here, we formulate a new physiology of pathological scarring based on the aberrant primary involvement of complement pathway components and discuss how this pathway may relate to a low expression of caveolin-1 (CAV1) in HTS and KE. Low level of CAV1 is currently recognized as a hallmark in inflammatory and hyperproliferative cutaneous conditions (8–10).

## Microbiota in wound healing and pathological cutaneous scarring

We appreciate that the majority of non-healing wounds are clinically infected and that the spatiotemporal behavior of microbiota in the wound is connected with healing outcomes (11). A retrospective study of 2963 samples from chronic wounds revealed that *S. aureus* was present in 63% of wounds of different ethiologies and that 25% of these wounds were contaminated with antibiotic-resistant *S. aureus* (12).

While the role of commensal and pathogenic microbiota in wound healing was intensively investigated, much less attention was paid to the microbial content in pathological scars. Recently, using the methods of 16S rRNA sequencing and metaproteomics, it was shown that HTS, compared to normal skin, have a dramatically increased content of inflammatory cells and modified microbiota, strongly dominated by Gram-positive bacteria such as *S. aureus* (13). The content of *S. aureus* in HTS was about 20 times increased *vs. C. acnes* which was about 90 times decreased compared to normal skin. Moreover, these modifications of *S. aureus*/*C. acnes* in HTS demonstrated a strong positive/negative correlation with the Vancouver Scar Scale, describing the clinical severity of HTS. *S. aureus* is the most common commensal pathogen found in the wound bed and is normally present at the site and time of skin injury (14). The low level of other commensal bacteria in HTS, such as *C. acnes*, can be explained by a dramatic reduction of the pilosebaceous units (PSU) in the scar tissue (which are the main sites for the location of these bacteria in the skin). In contrast, the appearance of *S. aureus* in mature HTS points to an aberrant innate immune response. Consistent with this model, mice kept in a germ-free environment demonstrate significantly accelerated and scarless wound healing, with strongly reduced content of TGF-β1, which clearly indicates the involvement of commensal microbiota both in the wound healing process and in pathological scarring (15). An increased content of *S. aureus* (about 33%) and decreased content of *C. acnes* (by about 2.5 times) was also reported in KE (16); however, these changes were not as dramatic as in HTS (13).

*S. aureus* impairs the functioning of dermal fibroblasts and demonstrates deleterious effects on wound healing (17). This pathogen significantly upregulates the transcription and translation of pro-inflammatory and pro-fibrotic molecules inducing murine mammary gland fibrosis (18) and is substantially involved in the development of cystic fibrosis (19).

Whereas *S. aureus* are Gram-positive bacteria, Gram-negative bacteria and their products can also significantly influence wound healing and scarring. Lipopolysaccharide (LPS) can effectively delay wound healing and increase skin scarring signaling pathways (20); even at low concentrations, it can transform normal skin fibroblasts into fibroblasts typical for HTS tissue (21). LPS is also well-known for its ability to induce innate immune reactions (22) and can promote skin scarring through activation of the TLR4 pathway in dermal fibroblasts (23). This is of special relevance for large surface burn wounds, which lead to increased gut permeability for endogenous pathogens and their products within a day after cutaneous damage (24), and thus can significantly increase LPS content in circulation.

## Skin protection against *S. aureus*

The skin possesses different defense options against *S. aureus*. The widespread presence of this pathogen in HTS indicates the malfunctioning of these defense mechanisms. This includes the activation of the complement pathway and the opsonization of bacterial membranes with complement factor C3b, i.e. the coating of bacterial surfaces with opsonins, which are soluble proteins that mark the cell for phagocytosis. Such opsonization is followed by the induction of the terminal complement cascade with the production of MACs in the plasma membranes. MACs can only lyse Gram-negative bacteria directly. In contrast, Gram-positive *S. aureus* displays a thick peptidoglycan layer, and *S. aureus* bacteria opsonized with C3b will be phagocytized by macrophages and neutraphils (25). This mechanism is however suppressed by C3 deficiency, whereas a reduction in C3 can be induced by *S. aureus* directly through the production of the protease aureolysin which can cleave C3 (26, 27). Not just aureolysin, but also other major extracellular proteases of *S. aureus* can potently inhibit complement pathway (28). Additionally, *S. aureus* can recruit CFH to its surface to inhibit the complement pathway (29). This provides an effective suppression of the CFH levels in the wound. This CFH reduction is sufficient to impact the fibrotic behavior of the healing wound (7). Together with C3b and properdin, CFH is an important recognition molecule for the host-*vs.*-target recognition mechanism. As a result, its suppression within the wound significantly increases the autolysis of the host cells mediated by the activation of the complement pathway.

Skin cells also have another mechanism against *S. aureus* connected with the production of the anti-microbial peptide cathelicidin (CAMP, or LL-37 in humans) by keratinocytes or by dermal adipocytes during their reactive adipogenesis. Reactive adipogenesis is a quick and massive proliferation and differentiation of dermal preadipocytes into mature adipocytes. This provides a pronounced expansion of the dermal white adipose tissue (dWAT) located within the dermal-hypodermal interface (30). The high abundance of *S. aureus* in the mature HTS indicates that either the reactive adipogenesis is suppressed or, alternatively, *S. aureus* in wounds prone to formation of HTS are able to evade the impact of CAMP through enhanced secretion of aureolysin.

Thus, transformation of *S. aureus* from commensal to pathogenic bacteria in the wound can induce significant modification of the immune reaction at the site of skin injury and thus influence the scarring process.

## The complement pathway in fibrotic diseases

Generally speaking, the activation of the complement pathway is involved in the pathogenesis of a number of different fibrotic diseases. Enhanced local tissue expression of anaphylatoxins C3a/C5a and MACs can be seen in idiopathic pulmonary fibrosis (IPF) characterized by an excessive accumulation of extracellular matrix in the lung’s interstitium. A pharmacological or RNA interference-specific suppression of C3aR/C5aR arrested the progression of experimental bleomycin-induced lung fibrosis (31). The proteins C5-C9 involved in the formation of MACs are upregulated in IPF (32). Complement activation is also an important factor in the development of kidney fibrosis (33). An increased expression of MACs was recognized as an important pathophysiological pro-inflammatory and pro-fibrotic factor in the kidney (34). Increased expression of MACs induces the production of collagen, thereby promoting a pro-fibrotic process in the kidney (35). Enhanced expression of MACs and C5aR was detected also in skin lesions of patients with systemic sclerosis, whereas C5aR was found even in unaffected skin areas (36). The expression of MACs, revealed at low concentrations even in normal skin, is significantly increased in various inflammatory cutaneous conditions (37).

Complement factor D (CFD) plays a major role in the healing of the injured Achilles tendon, demonstrating a repair sequence similar to other tissues, including the skin; CFD is significantly downregulated in healing tendons compared to its contra-lateral healthy counterpart (38). Moreover, in a comparison of patients with good *vs.* poor healing outcomes, CFD expression is significantly higher in the inflammatory phase of healing and lower in the proliferative phase (39). CFD is the rate-limiting factor in the alternative complement pathway, catalyzing the formation of C3 convertase (C3bBb) by cleaving CFB; thus, elevated expression of CFD can lead to a reduction of C3 levels (40). On the opposite end of the spectrum, a reduced expression of CFD corresponds to an increased expression of C3, which directly influences the local production of collagen in affected skin areas. A downregulated expression of CFD during the remodeling phase of wound healing leads to an overproduction of collagen at the site of injury. This effect is reduced in aging skin characterized by an increased expression of CFD (41). This can explain the decreased incidence of keloids and HTS at older age after reaching its highest incidence level in the second decade of life (42).

Adiponectin (ADN) is a protein secreted by adipocytes that demonstrates anti-inflammatory effects, potently suppressing LPS-induced NF-kB activation and IL-6 expression in WAT (43). ADN displays a high structural similarity with some factors of the complement protein family. Some limited data suggest that it may activate the classical complement pathway through binding to C1q, thereby initiating the formation of MACs (44). We have demonstrated in a murine model that the expression of ADN and CAMP are significantly increased at the site of skin wounding, whereas the content of ADN in human keloids is reduced by several orders of magnitude compared to the normal control skin (45). Others have reported a strong negative correlation between the serum ADN levels and Vancouver Scar Scale scores in keloid patients (46). Moreover, human cathelicidin LL-37 inhibits both baseline and TGF-β-induced collagen expression. It demonstrates an inverse correlation with cutaneous fibrosis and is strongly reduced in keloids (47). This further lends support to the idea there is a deficiency of reactive adipogenesis in pathological cutaneous scars. Remarkably, reactive adipogenesis, which is a unique feature of dermal adipocytes, is reduced both in murine and human aging skin. This was primarily connected to a reduction in the number of dermal preadipocytes and is mediated by TGF-β (48).

In normal wound healing, COL3 is produced in the proliferative phase and is replaced by COL1 in the remodeling phase. COL3 forms a weak network serving as an elastic rebound, whereas COL1 provides tissue with high tensile strength. Analysis of HTS in different stages of their maturation at 6–12 months and 18–24 months after burning provided with immunohistochemistry and laser confocal microscopy did not reveal difference in COL1 levels, but a significantly increased content of COL3 in HTS compared to non-HTS scars (49). An enhanced production of COL3 can be induced through a deficiency of CFH (50) which regulates complement activation by interacting with the central complement protein C3b; CFH deficiency in HTS can be induced by pathogenic *S. aureus*, recruiting CFH to the bacterial surfaces (29). This suggests that *S. aureus* could be responsible for the shift of the COL1/COL3 ratio in HTS, a known hallmark of this skin pathology.

All of these observations indicate that different components of the complement system, especially CFD and CFH, participate in the formation of HTS, and highly likely also of KE. This is due to their involvement in the regulation of the amount and type of collagen produced in different phases of wound healing.

## DWAT and its role in HTS formation

DWAT (in humans also occasionally referred to as skin associated adipose tissue, SAAT) has a special geometry combining a thin superficial layer of dermal adipocytes adjacent to the dermal-hypodermal interface with typical cone-like structures concentrated around pilosebaceous units (PSUs) (51, 52). These cone-like structures which appear as protrusions of dWAT into the reticular dermis were earlier described as the prevalent sites of HTS formation (53). Murine lineage-tracing studies demonstrated that fibroblasts appearing in cutaneous lesions and fibrotic dermis are of adipogenic origin (54).

Dermal adipocytes demonstrate rapid phenotypical transformations, such as de-differentiation into fibroblast-like preadipocytes at the catagen phase and re-differentiation into mature adipocytes during the anagen phase of the hair follicle (HF) cycle. Moreover, some preadipocytes can leave this cycle and transdifferentiate into myofibroblasts, thereby enhancing collagen production (45, 55).

Considering that C3, CFB and CFD can be locally produced by adipocytes and that dermal cones can deeply penetrate in the dermis, dermal adipocytes likely serve as an important source of complement factors and cathelicidin in the wound. Thus, they can directly influence wound healing and HTS formation (45). Whereas a shallow cutaneous wound insult will not sufficiently damage dWAT, full-thickness burn injuries destroying the dWAT structure will lead to a strong reduction of CFD expression during the remodeling phase of wound healing and result in a reduction of reactive adipogenesis and thus promote the formation of cutaneous scars.

## A crucial role for caveolin-1 in wound healing and cutaneous fibrosis

CAV1 is causally involved in wound healing, but its roles in acute and chronic wounds are apparently different: CAV1 is upregulated in wound edges of chronic wounds but reduced in acute wounds, such as burns (56). The causal role of CAV1 in the formation of chronic wounds was demonstrated through pharmacological or genetic disruption of caveolae which sufficed to achieve restoration of wound closure (56). Since the main problem with cutaneous scarring is the healing of acute wounds (especially of full thickness burns), it is fair to assume that low CAV1 expression in these injuries may also be typical for HTS and KE.

Reduced expression of CAV1 was indeed recognized as an important pathophysiological factor in fibrosis and scarring (8, 57). However, the underlying mechanisms for this reduction are still not fully understood. CAV1 is the main structural component of caveolae and has widespread involvement in the regulation of cellular signaling and the processes of endo- and exocytosis. A high concentration of CAV1 in the plasma membrane induces an increased formation of caveolae that can trap and internalize TGF-β receptors by CAV1-dependent endocytosis, thus effectively downregulating collagen production (58). A downregulated CAV1 expression cannot support this type of regulation as it was recently shown in Dupuytren’s disease - another fibroproliferative pathology affecting palmar fascia (59). CAV1 expression is significantly reduced in HTS- and KE-derived human fibroblasts. This reduction induces fibrotic responses (60, 61). Low levels of CAV1 are not just an important contributing pathogenic factor for inflammatory skin conditions (9, 10, 62, 63), but are also typical for HTS. Additionally, cells deficient for CAV1 demonstrate an increased uptake of fibronectin-binding pathogens, such as *S. aureus* (64). The low CAV1 levels may therefore explain at least partly the dramatically increased frequency of *S. aureus* observed in HTS (13).

Low levels of CAV1 prompt the activation of the runt-related transcription factor 2 (RUNX2) involved in osteogenesis and chondrogenesis (57). Correspondingly, the enhanced expression of RUNX2 will promote the formation of the cartilage-like, hyalinized tissue that is typical in KE. H&E analysis of hyalinized areas in KE revealed a matrix structure typical for cartilage (65), which also suggests the involvement of RUNX2 in KE pathogenesis. Remarkably, overexpression of RUNX2 accelerates closure of the burn wounds (66).

The high presence of pathogens and the activation of the terminal complement pathway during the formation of HTS and KE indicates an increased propensity for MACs formation in these skin conditions. Increased MACs can cause not only the death of invading pathogens, but also prompt autolysis of host cells. To avoid this, the formation of MACs must be counteracted by cellular defense mechanisms, such as the expression of inhibitors blocking MAC assembly (such as CD59/protectin) as well as removal of opsonins from plasma membranes through shedding (involving the MMP14 axis) or by internalization and degradation of assembled MACs through endocytosis (22). Internalization of MACs can be achieved through CAV1/DNM2 (dynamin-2) (67) or clathrin/DNM2-dependent endocytosis (68). The former of the two pathways is dominated by the availability of CAV1. The role of this pathway is becoming even more important in the case of CFH deficiency, a state typical for fibrotic wounds (7). Thus, a typical CAV1 deficiency in inflammatory and fibrotic conditions reflects the activation of the terminal complement pathway and CFH deficiency, causing a widespread induction of the CAV1-dependent endocytosis pathways for MACs from the plasma membranes of the host cells to protect these cell from autolysis.

## A new pathophysiology of cutaneous scarring

**Figure 1:**
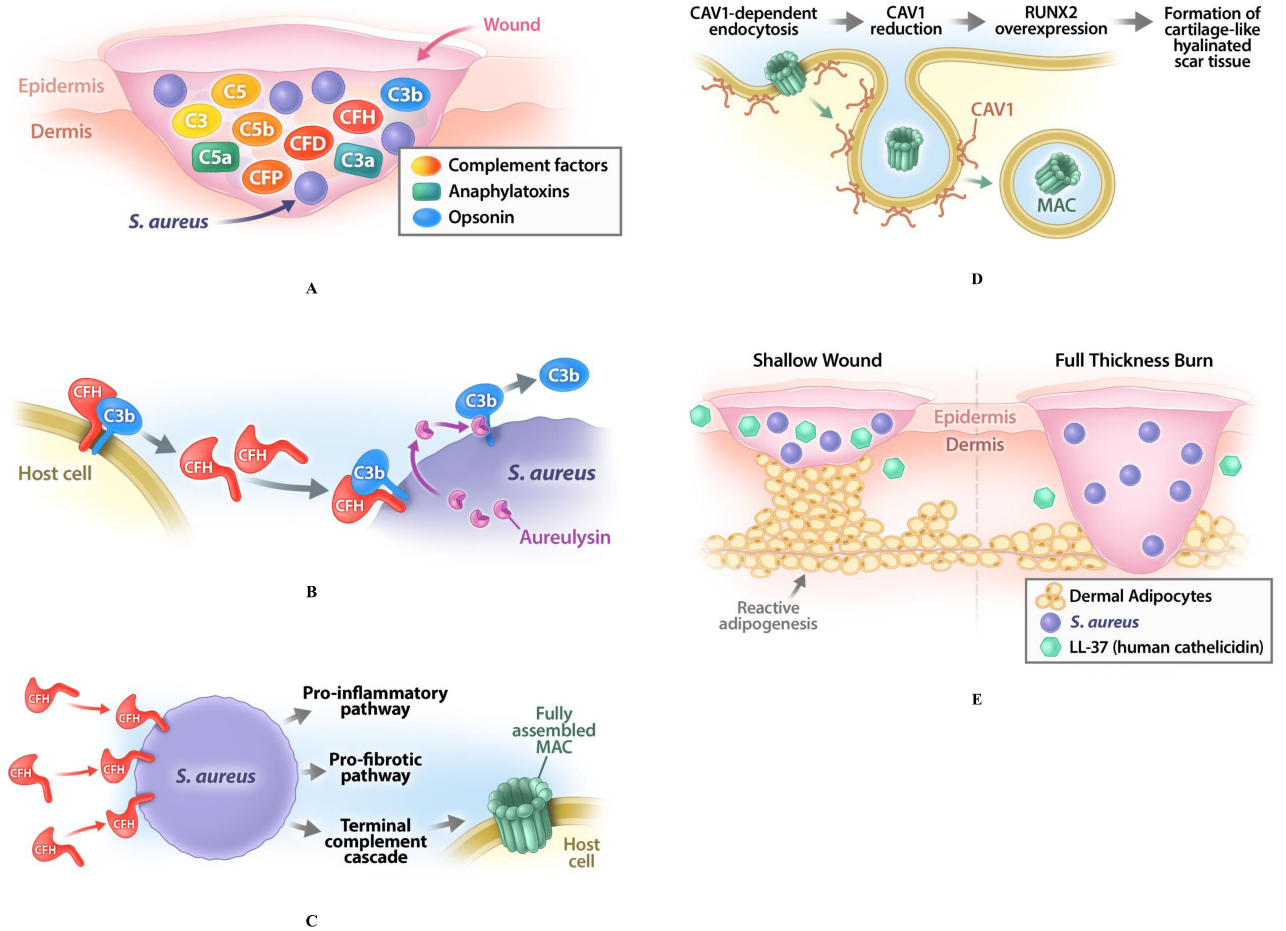
Pathophysiological model for pathologic cutaneous scarring. **(A)** Skin wounding induces the activation of the complement pathway to promote healing; this activation relates to production of different complement factors such as C3, C5, factor B (CFB), factor D (CFD), factor H (CFH), as well as of anaphylatoxins (C3a, C5a) and opsonins (C3b). **(B)** The commensal bacterium *S. aureus*, which is always present at the site and time of injury, will be opsonized by complement factor C3b, which forces these bacteria to produce aureolysin to remove C3b to avoid phagocytosis; this leads to widespread proliferation of *S. aureus* changing their status from commensal to pathogenic. **(C)** *S. aureus* recruit CFH to their surfaces, which leads to CFH deficiency in the wound. This deficiency impairs the host-or-target identification and increases the probability of the autolysis of the host cells through the production of the membrane attack complexes (MACs) in their membranes. Such CFH deficiency induces the overproduction of the COL3, thus increasing the COL3/COL1 ratio in fibrotic compared to the non-fibrotic wounds. **(D)** To counteract this process, defense mechanisms are activated in the injured area. One of them is the CAV1-dependent endocytosis, effectively removing the MACs from the plasma membrane; CAV1 recruitment for this process consistently leads to a low content of CAV1 in the affected skin area, providing a known hallmark of HTS and KE. Reduction of CAV1 leads to a dysregulation of cellular signaling and causes overexpression of RUNX2, which promotes the formation of the cartilage-like hyalinated scar tissue. **(E)** While pathogenic *S. aureus* normally activates also another defense mechanism - reactive adipogenesis in dermal white adipose tissue (dWAT) and production of cathelicidin (LL-37) - this activation is absent in full-thickness burns with a destroyed dWAT layer, which allows *S. aureus* to survive and colonize the subsequently formed scars.

Based on the observations outlined above, we synthesize the following pathophysiological model for pathologic cutaneous scarring (**Figure 1**).

1. Skin wounding induces the activation of the complement pathway to promote healing.
2. The commensal bacterium *S. aureus*, which is always present at the site and time of injury, will be opsonized by complement factor C3b, which forces these bacteria to produce aureolysin to remove C3b to avoid phagocytosis; this leads to widespread proliferation of *S. aureus* changing their status from commensal to pathogenic.
3. *S. aureus* also recruit CFH to their surfaces to evade the humoral immune system, which leads to CFH deficiency in the wound. This deficiency impairs the host-or-target identification and increases the probability of the autolysis of the host cells through the production of MACs in their membranes. Additionally, CFH deficiency induces the overproduction of the COL3, thus increasing the COL3/COL1 ratio in fibrotic compared to the non-fibrotic wounds.
4. To counteract this process, defense mechanisms are activated in the injured area. One of them is the CAV1-dependent endocytosis, effectively removing the MACs from the plasma membrane; CAV1 recruitment for this process consistently leads to a low content of CAV1 in the affected skin area, providing a known hallmark of HTS and KE. Reduction of CAV1 leads to a dysregulation of cellular signaling and causes overexpression of RUNX2, which promotes the formation of the cartilage-like hyalinated scar tissue.
5. While pathogenic *S. aureus* normally activates also another defense mechanism - reactive adipogenesis in dWAT and production of cathelicidin - this activation is absent in full-thickness burns with a destroyed dWAT layer, which allows *S. aureus* to survive and colonize the subsequently formed scars.

Remarkable correlations between the state of the complement pathway and CAV1 content in the injured skin, especially the low level in acute and high levels in chronic wounds, the possibility to accelerate wound closure through activation of complement/CAV1 in acute wounds and to induce this closure through their suppression in chronic wounds as well as the reduced values of CAV1 observed in HTS and KE indicate that proper regulation of CAV1 during early stages of acute skin injury can serve as a target for prevention of pathologic cutaneous scarring.

This pathophysiology that takes into consideration the aberrant behavior of the complement pathway during wound healing as a central point for pathological cutaneous scarring will need further experimental verification in future research. According to this pathophysiology, future efforts in the prevention and treatment of scarring should be concentrated on the reduction of bacterial content in the wound, in combination with a proper modulation of the complement pathway and CAV1 in the wounded skin.
